# Activity Preferences Among Older People With Dementia Residing in Nursing Homes

**DOI:** 10.3389/fpsyg.2021.799810

**Published:** 2022-01-20

**Authors:** Eun-Young Park, Jung-Hee Kim

**Affiliations:** ^1^Department of Secondary Special Education, College of Education, Jeonju University, Jeonju, South Korea; ^2^College of Nursing, The Catholic University of Korea, Seoul, South Korea

**Keywords:** decision tree, older people, dementia, nursing homes, activity preferences

## Abstract

The study aimed to examine the influence of personal characteristics on activity preferences using decision tree analysis and examine the effects of the variables using conventional approaches (logistic regression analysis). A descriptive study was conducted with 251 nursing home residents with dementia in Korea (76.9% female) to examine the relationship between their personal characteristics and activity preferences. Decision tree analysis was used to classify participants’ activity preferences, and preference levels were examined using logistic regression analysis. Activities were classified as either physical and social activities or cognitive and affective activities. This model showed an accuracy rate of 85.7% for positively predicting physical and social activity preference and 30.3% for positively predicting cognitive and affective activity preference. Gender was the strongest determinant of activity preference. The odds of preferring physical and social activities were 3.179 times higher among women, while the odds for preferring cognitive and affective activities were 0.412 times higher among men. Notably, cognitive and affective activity preference increased to 58.8% for married male participants. This study’s findings can contribute to the development of programs to decrease behavioral and psychological symptoms among older people with dementia residing in nursing homes and provide scientific evidence for integrating these activities into long-term services for this population.

## Introduction

A major problem related to dementia is cognitive decline, which makes it difficult for people to maintain their usual activities (Nygård, 2004; Van Der Roest et al., 2007; O’Sullivan and Hocking, 2013). People with dementia tend to be less involved in leisure activities due to the loss of their physical and cognitive abilities, which could lead to feeling isolated from friends and family, depressive mood, and boredom (Engelman et al., 1999; Feliciano et al., 2009). In particular, boredom and loneliness are common among people with dementia who reside in nursing homes (Cohen-Mansfield et al., 1992, 2017). Several studies have reported that environmental stimuli can increase behavioral and psychological symptoms and decrease positive affect among people with dementia (Cohen-Mansfield and Werner, 1997; Cohen-Mansfield et al., 2010a,^2011^). Further, various activities, including physical activities, have the potential to improve the quality of life of people with severe mental health disorders (Richardson et al., 2005b).

Patients with dementia prefer positive stimuli and close social relationships over superficial encounters with institutional staff during their daily treatment (Mark, 2012). Socioemotional selectivity is a theory of life-span development grounded in the uniquely human ability to monitor time. The preferences, social networks, and emotional experiences of patients with dementia could be informed by the theory of life-span development. The emphasis on individual strengths and personal resilience is likely to be especially appealing to older people (Carstensen, 2021). Patients with cognitive impairments also have normal human psychological needs, including comfort, identity, attachment, occupation, and inclusion (Kitwood, 1997). Strategies for accessing the internal experiences of patients with dementia may be useful to understand them as individuals. However, only limited knowledge is available about the experience of those with dementia due to the deleterious effects of the stereotypes regarding individuals afflicted with it (Mast, 2009).

Both personal and group activities, including those involving families, have been found to be important for people with dementia (Chester et al., 2018; Roberts et al., 2018). Moreover, maintaining social contact and participating in family activities could increase older people’s life satisfaction and self-esteem and their perception of having continuity across their life course (Vikström et al., 2008; Cruz et al., 2013; Jøranson et al., 2016; Olsen et al., 2019). Physical activity interventions for those with mental health disorders must be tailored to individual preferences, which can also be beneficial for leisure and activities of daily living (Richardson et al., 2005a). For patients with dementia, activity is perceived as important, and while person-centered approaches are expected to contribute to activity promotion, individualized activities are often not provided for these patients (Van’t Leven et al., 2018).

According to Cohen-Mansfield et al. (2017), contributing factors that affect activity participation can be categorized as personal, environmental, or stimulus. Dementia patients’ endorsed activities were affected by gender and physical or cognitive function. Individualized support should be provided considering personal preferences and characteristics, such as stage of dementia, physical function, and health status (Ablitt et al., 2009; Peeters et al., 2010; Van Mierlo et al., 2010). These activity preferences were also affected by other personal factors, such as past experiences (Cohen-Mansfield, 2017). In addition, patients’ needs may be impacted by culture, and health care professionals need to show continuous cultural sensitivity to patients’ needs. This information is important for planning activities and intervention programs that are closely related to endorsement of or engagement in activities. However, few studies have examined the activity preferences of older people with dementia residing in nursing homes. Decision trees based on real-word data have been used to create rules for activity preferences, as they can detect previously unknown interactions among various items of clinical information and reveal relationships between assessment outcomes and patient characteristics. This technique can identify data patterns that distinguish between preferred activities and other activities, revealing potentially complex relationships among individual characteristics (Myles et al., 2004).

A preference assessment could guide health care professionals in providing reinforcements to patients to increase the future probability of a behavior. A preference assessment is defined as a process to identify reinforcers that will motivate an individual at a specific point in time (Chen and Chen, 2005). Identifying factors that may function as positive stimuli and environments is important, as results of previous research indicate that a tailored intervention is more efficacious than treating patients as a homogenous group (Vollmer et al., 1994). Although there is a substantial need to assess activity preferences among patients with dementia, limited cognition and a lack of verbal responsiveness can be barriers to fully discerning their preferences (Mast, 2009).

This descriptive study examined personal characteristics within the context of preferred activities among nursing home residents with dementia. The study aimed to examine the influence of personal characteristics on activity preferences using decision tree analysis and examine the effects of the variables using conventional approaches (logistic regression analysis).

## Methods

A secondary analysis was conducted that aimed to identify the activity preferences of older people with dementia residing in nursing homes in Korea.

### Data

The source of the data was a large-scale research project which focused on the behavioral and psychological symptoms of patients with dementia to develop an intervention program to improve their quality of life. The project obtained approval from the Institutional Review Board of Catholic University of Korea (MC18QNSI0055). Details of the sampling process have been reported elsewhere (Park et al., 2019). Data were collected from six nursing homes selected at random in the Seoul and Gyeonggi regions of Korea after explaining the purpose of the study and obtaining approval from the directors and nurse managers. Participants were recruited by an ad posted in the approved facilities for 2 weeks, and patients willing to participate in the study who met inclusion criteria were selected. When older people with dementia expressed their willingness to participate, a mental health expert at the facility confirmed the patient’s ability to consent. If they agreed, the researchers then contacted the participants and their legal guardians to obtain written consent.

The researchers surveyed the patients in person upon consent from the patient and their family. The inclusion criteria were being 65 years of age or older with a diagnosis of dementia, a resident or daycare visitor with dementia, and ability to respond to questions about activity preferences. The exclusion criteria included having other mental disorders, neurological disorders, or metabolic disorders. Among the 325 recruited participants, 70 participants who either did not report preferred activities or whose caregivers did not report such activities on their behalf were excluded. Only 4 participants were excluded due to having another diagnosed mental disorder. Ultimately, 251 participants were included and 74 participants were excluded from the study.

The minimum data size required for classification matrix research using data mining is calculated as follows: 6 × number of groups for the output variable × number of variables (Delmater and Hancock, 2001). For the number of groups for the output variable, the preferred activities among patients with dementia were classified into two types, and there were seven variables (gender, age, education level, marital status, religion, use of assistive devices, and ability for self-expression), which resulted in a minimum data size of 140.

### Data Collection

Data were collected by trained researchers from medical records or by interviewing patients with dementia regarding gender, age, education level, marital status, religion, use of assistive devices, and ability for self-expression. To examine patients’ preferred activities, an open-ended question was used: “What kind of activities do you like to do?” If the patients could not answer the question, their caregiver, who had provided care for the patient for at least 4 weeks, answered for them as a proxy. The question about the ability to express one’s opinions to others was also answered by the caregiver if the patient could not answer. A trained researcher asked the patients the questions directly using a structured questionnaire and recorded their responses.

The recorded activities were classified according to the criteria described below. Activities such as taking a stroll, participating in activities offered at the facility, talking to people, participating in religious activities, meeting with family, and talking to someone on the telephone were classified as physical and social activities (activities usually involving other people). Activities such as watching television, playing traditional games, doing puzzles, reading picture books, drawing, and singing were classified as cognitive and affective activities (activities usually performed alone). Two independent researchers (A and B) performed the classification.

### Statistical Analysis

A decision tree analysis was performed in this study. A decision tree is a data mining technique that explores, identifies, and models relationships, patterns, and rules within a dataset. Decision tree analysis graphs decision-making rules in a tree-like structure and performs classification and prediction. One of the benefits of decision tree analysis is that it expresses the processes of classification and prediction through induction rules according to the tree structure; thus, it is easier for the user to understand these processes compared to neural network analysis, discriminant analysis, or regression analysis (Kim et al., 2006). A decision tree can be used to identify the variables needed for analyses, such as discriminant analysis or regression analysis, and the interaction effects to be included in the model, and a decision tree itself can be used as a classification or predictive model (Ablitt et al., 2009). A decision tree features a tree structure consisting of nodes.

Decision tree analysis was performed according to the following steps. First, a tree structure was formulated by designating the appropriate split criterion and stopping rule according to the purpose of the analysis and data structure. In this study, as the target attribute was discrete, splitting occurred based on the frequency of data for each category of the target attribute, based on which a classification tree was built. The stopping rule was set to maximum tree depth = 5, minimum number of cases for parent node = 30, and minimum number of cases for child node = 10. To develop an ideal model using data mining, it is desirable to create various predictive models from a single dataset and comparatively analyze them (Song and Ying, 2015). Thus, the entire dataset was divided into training data and test data, and a model was created using the training data and verified using the test data. Hence, in this study, the ratio of training data to test data was set to 1:1.

For the decision tree analysis, the most universally used Classification and Regression Trees (1984; CART) algorithm was applied. The relationships between the characteristics of patients with dementia and their activity preferences were analyzed using logistic regression. SPSS 25.0 software was used for statistical analysis.

The dependent variable was the preferred activity, and it was divided into physical and social activities and cognitive and affective activities for analysis. Predictors could be divided into (a) demographic characteristics and (b) ability outcomes. Demographic characteristics included gender, age, education level, marital status, and religion, and ability outcomes included the use of assistive devices for mobility and ability for self-expression, defined as the ability to express one’s opinions to others. Marital status was recorded as either married or not married. Individuals were considered not married if they were widowed, single, or divorced. Religion was coded as either “yes” or “no,” and the use of assistive devices for mobility referred to the use of devices such as crutches, wheelchairs, or canes.

## Results

### Participant Characteristics

A total of 251 participants were included in the analysis. The sample was 76.9% female and 22.7% male, and 49.4% were aged 85 years or older. Of the participants, 72.5% were not married and 26.3% were religious. The most common education level was middle school graduation or lower (59.0%), and 22.7% of the participants used an assistive device for mobility. Each participant’s level of self-expression was recorded as either high (63.7%) or low (31.5%) (Table 1).

**TABLE 1:** Characteristics of patients with dementia (*n* = 251).

Characteristics	n	%
**Gender**		
Male	57	22.7
Female	193	76.9
Missing	1	0.4
**Age**		
Below 84	122	48.6
Above 85	124	49.4
Missing	5	2.0
**Presence of spouse**		
Yes	69	27.5
No	182	72.5
**Religion**		
Yes	66	26.3
No	185	73.7
**Education level**		
Below middle school	148	59.0
Above middle school	90	35.9
Missing data	13	5.2
**Use of assistive device**		
No	184	73.3
Yes	57	22.7
Missing data	10	4.0
**Degree of self-expression**		
High	160	63.7
Low	79	31.5
Missing data	12	4.8

### Predictors of Physical and Social Activity Preference

**FIGURE 1:**
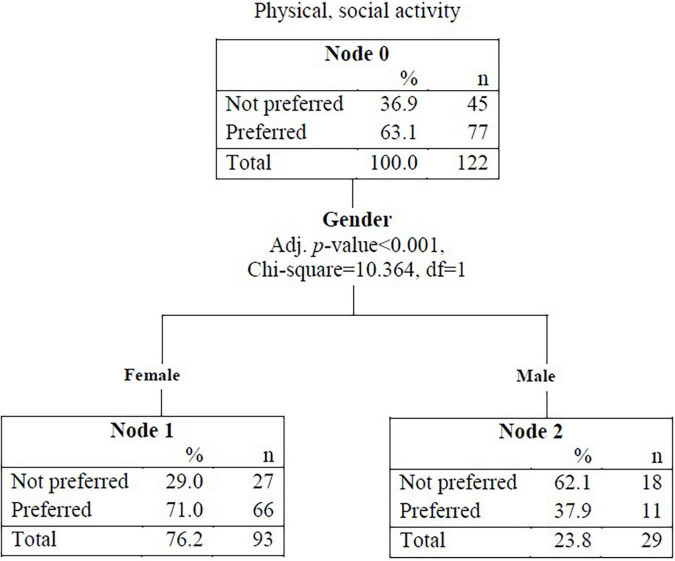
Decision tree for predicting physical and social preference.

The results of the decision tree used to predict physical and social activity preference among participants are illustrated in Figure 1. The strongest discriminant of physical and social activity preference was patient gender. Specifically, 63.1% of the entire sample preferred physical and social activities; however, when participants were stratified by gender, this increased to 71.0% for female participants and decreased to 37.9% for male participants (Figure 1).

This model showed an accuracy rate of 40.0% for predicting those who did not prefer physical and social activities (18 out of 45 patients) and an accuracy rate of 85.7% for predicting those who did prefer physical and social activities (66 out of 77) (Table 2). The influence of patient characteristics such as gender, age, education level, marital status, self-expression, use of assistive devices, religion, and children are shown in Table 3. Gender was identified as a significant predictor of physical and social activity preference [*B* = 1.157, S.E. = 0.388, Exp (β) = 3.179, *p* = 0.003]. The odds of preferring physical and social activities were 3.179 times higher among women than men.

**TABLE 2:** Classification matrix of CHAID for physical and social activities.

Classification matrix		Prediction			Forecasting	
		Not preferred	Preferred	Total	Accuracy measures	%
Training data	Not preferred	18	27	45	Specificity	40.0
	Preferred	11	66	77	Sensitivity	85.7
	Total	29	93	122	Overall accuracy	68.9

*CHAID, Chi-squared automatic interaction detection.*

**TABLE 3:** Summary of logistic regression analysis for the preference of physical and social activities.

Variable	B	S.E.	df	Exp (β)	–95% CI	+95% CI	*p*
Gender^a^	1.16	0.39	1	3.18	1.49	6.80	0.003
Age^b^	–0.06	0.31	1	0.94	0.51	1.73	0.834
Education level^c^	–0.11	0.32	1	0.90	0.48	1.69	0.735
Presence of spouse^d^	–0.32	0.36	1	0.73	0.36	1.46	0.372
Degree of self-expression^e^	0.37	0.33	1	1.45	0.76	2.76	0.261
Use of assistive device^f^	–0.65	0.34	1	0.52	0.27	1.02	0.056
Religion^g^	–0.17	0.34	1	0.84	0.43	1.64	0.614
Children^h^	1.51	0.83	1	4.54	0.90	22.99	0.068

*^a^Dummy variables (Reference = female).*

*^b^Dummy variables (Ref = above 85).*

*^c^Dummy variables (Ref = below middle school).*

*^d^Dummy variables (Ref = no).*

*^e^Dummy variables (Ref = low).*

*^f^Dummy variables (Ref = no).*

*^g^Dummy variables (Ref = no).*

*^h^Dummy variables (Ref = no).*

### Predictors of Cognitive and Affective Activity Preference

**FIGURE 2:**
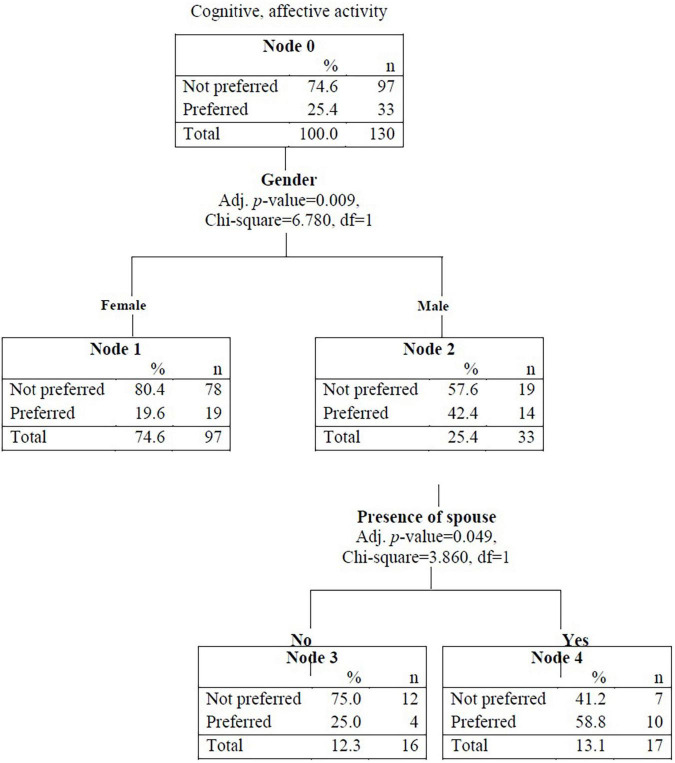
Decision tree for predicting preference in cognitive and affective activities.

The results of the decision tree for predicting cognitive and affective activity preference are illustrated in Figure 2. The most potent discriminant of cognitive and affective activity preference was patient gender. Specifically, 25.4% of the entire sample preferred cognitive and affective activities; however, when stratified by gender, this decreased to 19.6% for female participants and increased to 42.4% for male participants. The second discriminant was marital status. The preference for cognitive and affective activity increased to 58.8% among male participants with a spouse. This model showed an accuracy rate of 92.8% for predicting those who did not prefer cognitive and affective activity (90 out of 97) and an accuracy rate of 30.3% for predicting those who did prefer cognitive and affective activities (10 out of 33) (Table 4).

**TABLE 4:** Classification matrix of CHAID for cognitive and affective activities.

Classification matrix		Prediction			Forecasting	
		Not preferred	Preferred	Total	Accuracy measures	%
Training data	Not preferred	90	7	97	Specificity	92.8
	Preferred	23	10	33	Sensitivity	30.3
	Total	113	17	120	Overall accuracy	76.9

*CHAID, Chi-squared automatic interaction detection.*

The influence of patient characteristics, such as gender, age, education level, marital status, self-expression, use of assistive device, religion, and children, on cognitive and affective activity preference are shown in Table 5. Gender [*B* = –0.886, S.E. = 0.405, Exp (β) = 0.412, *p* = 0.029] and education level [*B* = 0.797, S.E. = 0.354, Exp (β) = 2.219, *p* = 0.024] were identified as significant predictors of cognitive and affective activity preference. The odds for preferring cognitive and affective activities were 0.412 times higher among men than women and 2.219 times higher among middle school graduates or higher than their less educated counterparts.

**TABLE 5:** Summary of logistic regression analysis for preference of cognitive and affective activities.

Variable	B	S.E.	df	Exp (β)	–95% CI	+95% CI	*p*
Gender^a^	–0.87	0.41	1	0.41	0.19	0.91	0.029
Age^b^	0.51	0.36	1	1.66	0.82	3.36	0.160
Education level^c^	0.80	0.35	1	2.22	1.11	4.44	0.024
Presence of spouse^d^	–0.30	0.38	1	0.74	0.36	1.55	0.425
Degree of self-expression^e^	0.01	0.36	1	1.01	0.50	2.05	0.982
Use of assistive device^f^	–0.11	0.38	1	0.90	0.43	1.89	0.771
Religion^g^	0.49	0.40	1	1.63	0.75	3.53	0.220
Children^h^	–0.16	0.76	1	0.86	0.19	3.76	0.835

*^a^Dummy variables (Reference = female).*

*^b^Dummy variables (Ref = above 85).*

*^c^Dummy variables (Ref = below middle school).*

*^d^Dummy variables (Ref = no).*

*^e^Dummy variables (Ref = low).*

*^f^Dummy variables (Ref = no).*

*^g^Dummy variables (Ref = no).*

*^h^Dummy variables (Ref = no).*

## Discussion

This study aimed to examine the influence of personal characteristics on activity preferences using decision tree analysis and examine the effects of personal characteristics using logistic regression analysis. Engaging in activities alleviates boredom among patients with dementia residing in nursing homes and helps to evoke positive emotions (Cohen-Mansfield et al., 2010a,^2011^).

The first predictor of activity preference was gender. In this study, older women were found to prefer physical and social activities, while older men were found to prefer cognitive and affective activities. Caregiving, which is typically considered to be a role for women, strengthens their interactions with others. Furthermore, women tend to be more relationship-oriented than men (Boyle, 2017), and thus seem to have demonstrated a higher preference for social activities. Other studies have documented that older women with dementia prefer cooking or baking (Menne et al., 2012; Cohen-Mansfield et al., 2019). Owing to the perception of traditional gender roles, in which women are expected to engage in housework and caregiving (Kyungsoon Park, 2018), activity preferences may differ between genders. Gender roles are learned over a prolonged period, and may therefore influence patients with dementia (Boyle, 2017). Further, older people with dementia have been reported to show consistent leisure activity preferences from the past to the present (Lepper et al., 2020) and more actively participate in activities when given a similar simulation to what they were used to in the past (Cohen-Mansfield et al., 2010b; Lopes et al., 2016). Thus, gender-specific activities should be planned for older people in nursing homes.

Despite the fact that men are traditionally seen as being engaged in physical labor (Kyungsoon Park, 2018), older men showed a low preference level for physical and social activities. This may be related to the characteristics of patients with dementia. In general, weakened physical functioning leads to patients with dementia being more sedentary than the general population, and studies have reported that walking, as opposed to more vigorous activities, is the most common physical activity among these patients (Daumit et al., 2005; Richardson et al., 2005b). Previous research reported that older women with dementia score various activities as more important than their male counterparts (Roberts et al., 2018), which supports our findings. A lack of periodic social contact is correlated with physical inactivity and lethargy (Daumit et al., 2005; McDevitt et al., 2006) and low self-efficacy (Trost et al., 2002). Thus, programs that promote physical and social activities in older men with dementia are also needed.

The higher preference for cognitive and affective activities among men in this study is similar to previous findings that men demonstrated a higher preference for games such as brain games (Ivory, 2006) and that older men like games (Menne et al., 2012; Cohen-Mansfield et al., 2019). A prior study also found that older men with dementia tend to be more individualistic and logical compared to their female counterparts (Boyle, 2017). Further, our results showed that the preference for cognitive and affective activities increased to 58.8% among older men with a spouse. According to Roberts et al. (2018), married individuals prefer activities such as bonding with family more than unmarried individuals; thus, continued attachment with a spouse seems to increase individuals’ preferences for these activities.

Multiple regression analysis confirmed that the odds of preferring cognitive and affective activities were higher among more highly educated individuals when adjusting for age, marital status, ability for self-expression, use of assistive devices, religion, and children. Education level seems to have affect preference levels, as cognition-based activities stimulate overall cognitive function through games, maps, and discussions and include concentration, memory, and problem-solving ability training (Kim, 2019).

In this study, we determined the hierarchy of personal characteristics of activity preferences using decision tree analysis and the effects of personal characteristics using logistic regression analysis. There is a difference between the data mining tools used for classification and logistic regression analysis. The logistic regression model serves to determine which variables predict treatment status and contribute to predicting preferred activities (Chatterjee and Hadi, 2015). However, data mining algorithms find the best fitting model through automated processes that search through the dataset to detect patterns. These patterns may include interactions between variables, as well as interactions within subsets of variables (Linden and Yarnold, 2016).

This study examined the relationship between personal characteristics and activity preferences of patients with dementia. The results of this study provide evidence to support the need for patients with dementia to be involved in activities and for nursing homes to provide these patients with a variety of activity programs. A tailored intervention program should be designed to meet the dementia patient’s preferences by securing their emotional immersion and engagement in accordance with their gender and marital status. Also, subsequent studies should also assess other psychosocial variables found to have a key role in activity involvement, such as self-efficacy, perceived social support, motivation, and pleasure.

This study’s findings can contribute to the development of programs to decrease the behavioral and psychological symptoms among older people with dementia residing in nursing homes and provide scientific evidence for integrating these activities into long-term services for this population.

Future research should explore valid methods to confirm the preferences of patients with cognitive impairments. As incomplete questionnaires were excluded from the analysis, it will be necessary to investigate the preferred activities of patients with relatively high cognitive levels and high activity, for example, patients with mild cognitive impairment. As reliability between proxy responses and those of patients with dementia were not confirmed, these methodological issues may have affected the interpretation of our data. Additionally, more long-term studies are needed to examine the relationship between these variables because the activity itself may be important, regardless of cognitive function and age (Roberts et al., 2018).

This study has some limitations. As this was a cross-sectional study, prospective studies with larger patient samples that consider various patient subgroups are needed in the future. In addition, we used caregivers’ responses for some measures and did not examine actual attendance duration and engagement in activities among patients with dementia. Because our study excluded the participants who did not report preferred activities and those who were diagnosed with other mental disorders, there are limitations regarding generalization to patients with dementia residing in nursing homes.

Nevertheless, we classified the activity preferences of older people with dementia residing in nursing homes using a decision tree and examined their preference levels through logistic regression analysis. Decision trees provided an effective method of decision making because it allowed us to create rules for activity preferences. Thus, information about patients’ activity preferences was useful for predicting whether they will commit to an activity and may offer knowledge relevant to providing person-centered care.

## Data Availability Statement

The original contributions presented in the study are included in the article/supplementary material, further inquiries can be directed to the corresponding author.

## Ethics Statement

The studies involving human participants were reviewed and approved by the Institutional Review Board of Catholic University (MC18QNSI0055). The patients/participants provided their written informed consent to participate in this study.

## Author Contributions

J-HK contributed to the conception of the study, interpretation of the data, and drafted the manuscript. E-YP conducted the statistical analyses and interpreted the data. Both authors read and approved the final manuscript.

## Conflict of Interest

The authors declare that the research was conducted in the absence of any commercial or financial relationships that could be construed as a potential conflict of interest.

## Publisher’s Note

All claims expressed in this article are solely those of the authors and do not necessarily represent those of their affiliated organizations, or those of the publisher, the editors and the reviewers. Any product that may be evaluated in this article, or claim that may be made by its manufacturer, is not guaranteed or endorsed by the publisher.
